# Metabolic syndrome in adult population of rural Wardha, central India

**Published:** 2010-12

**Authors:** Pranita Kamble, Pradeep R. Deshmukh, Neelam Garg

**Affiliations:** *Department of Biochemistry, Mahatma Gandhi Institute of Medical Sciences, Sewagram, India*; * *Dr Sushila Nayar School of Public Health, Mahatma Gandhi Institute of Medical Sciences, Sewagram, India*

**Keywords:** ATP-III, dyslipidaemia, metabolic syndrome, modified ATP-III

## Abstract

**Background & Objectives:**

Prevalence of metabolic syndrome is high among Asians including Indians. Scarce information is available about the magnitude of metabolic syndrome in rural areas and hence present study in rural area of Wardha district, central India.

**Methods:**

In 300 randomly selected subjects, blood pressure and anthropometric measurements such as height, weight, waist circumference and hip circumference were noted. Blood sample was collected after overnight fasting and was subjected to biochemical quantification such as fasting blood sugar, total cholesterol, triglycerides, HDL-C, VLDL-C and LDL-C. Data were analyzed using ATP-III definition as well as by modifying the waist circumference cut-offs as per Asia-Pacific guidelines.

**Results:**

Overall metabolic syndrome as per ATP-III criteria was observed in 5.0 per cent adult rural population. When ATP-III criteria were modified using waist circumference cut-offs recommended by Asia-Pacific guidelines, metabolic syndrome was seen in 9.3 per cent. It was 10.7 per cent among females and 8.2 per cent among males. Receiver operating characteristic curve was plotted to find out the best cut-off of BMI to identify the individuals with metabolic syndrome. The best cut-off for BMI came out to be 23.32 kg/m^2^.

**Interpretation & conclusions:**

The magnitude of metabolic syndrome was low among rural adults of Wardha as compared to reported values in urban areas. BMI of 23.32 kg/m^2^ and higher was found to predict significant risk of metabolic syndrome in these study subjects. However, studies with larger sample need to be conducted to confirm these findings.

Metabolic syndrome is a constellation of risk factors such as central obesity, increased blood pressure, impaired glucose tolerance, altered lipid profile mainly low high density lipoproteins (HDL) and high triglycerides which predispose the individual to increased risk for development of diabetes mellitus and cardiovascular diseases^1^,^2^. Its pathogenesis is complex, but interaction of obesity, sedentary lifestyle and dietary and genetic factors are known for their contribution^2^,^3^. It is a useful tool to identify individuals at risk for diabetes and coronary heart disease. Epidemiological studies have reported a high prevalence of metabolic syndrome and cardiovascular mortality among non-resident Indians settled abroad^4^–^6^. Lifestyle factors appear to play an important role as obesity especially abdominal obesity, and dyslipidaemia worsen with urbanization and migration^7^,^8^.

Prevalence of metabolic syndrome is high among Asians including Indians, and is rising, particularly with the adoption of modernized lifestyle. Many studies in India have reported high prevalence of metabolic syndrome^9^–^11^. But these studies were carried out mainly in urban areas. Scanty information is available about the magnitude of metabolic syndrome in rural areas. Hence the present study was undertaken in rural area of Wardha district, central India, to find out the magnitude of metabolic syndrome.

## Material & Methods

### 

#### Study setting and study subjects:

The present cross-sectional study was carried out in all the villages of Primary Health Center (PHC), Anji from February 2007 to February 2008. The study site was located in rural Wardha district, about 758 km east from State capital Mumbai. Anji PHC covers 23 villages with a population of 33000 and is a field practice area of Mahatma Gandhi Institute of Medical Sciences, Sewagram. All adult residents of these 23 villages (above the age of 18 yr) were included in sampling frame. The protocol was approved by the institutional ethical committee.

#### Sampling method and sample size:

Considering 10 per cent prevalence of metabolic syndrome 3 per cent absolute error, alpha=0.1 and 10 per cent non-response; sample size required was 300^8^. The study subjects were selected by simple random sampling using computer generated numbers. The written informed consent was obtained from each participant.

#### Anthropometric measurements:

Body weight, height, waist and hip circumference were measured^12^. Body mass index (BMI) was calculated as weight in kilograms divided by squared height in meter.

#### Laboratory analysis:

The previous evening, the participants were visited and written informed consent was obtained after explaining the objectives and procedures of the study. After overnight fast blood samples (10 ml) were collected. The fasting blood sugar was analyzed by glucose oxidase peroxidase (GOP-PAP) method^13^; total cholesterol was analyzed by CHOD-PAP method^14^; triglycerides by GPO-PAP Trinder method^15^; and HDL-C by phosphotugstic acid method^16^. VLDL-C was calculated by indirect method as VLDL cholesterol is one-fifth of triglyceride level^17^. LDL-C was calculated by subtracting VLDL-C and HDL-C from total cholesterol. ERBA kits supplied by Transia Biochemicals Ltd., Mumbai, were used.

#### Statistical analysis:

According to the National Cholesterol Education Program Adult Treatment Panel (ATP) III,^18^the diagnosis of metabolic syndrome was made when three or more of the following risk factors were present: a waist circumference >102 cm in men and >88 cm in women, fasting glucose ≥110 mg/dl (6.1 mmol/l), systolic blood pressure ≥130 mmHg or diastolic blood pressure ≥ 85 mmHg, fasting triglycerides ≥150 mg/dl (1.7 mmol/l), and HDL cholesterol < 40 mg/dl (1.0 mmol/l) in men and < 50 mg/dl (1.3 mmol/l) in women. ATP III definitions were based on the association of factors with subsequent coronary heart disease in Caucasian cohorts. As Indians have higher body fat content than their western counterparts for the same BMI, lower cut-offs of waist circumference were used as suggested by Asia-Pacific guidelines^19^. Waist circumference (WC) cut-offs were taken as >90 cm for males and >80 cm for females to define overweight^19^.

Chi-square test was used to test the null hypothesis. All *P* values were two-tailed and statistical significance was defined as *P*<0.05. Receiver operating characteristic (ROC) analysis was carried out to identify the optimal cut-off for the BMI to identify the individuals with metabolic syndrome.

## Results

Of the 300 subjects selected, 101 (33.7%) were in the age group of 18-30 yr, 119 (39.6%) were in the age group of 31-50 yr while 80 (26.7%) were above 50 yr. Majority of the study subjects (56.3%) were males (Table I).

**Table I:** Age and sex distribution of the study subjects

Age (yr)	Sex		Total Number (%)
	Male Number (%)	Female Number (%)	
18-30	51 (50.5)	50 (49.5)	101 (33.7)
31-50	64 (53.8)	55 (46.2)	119 (39.6)
51 and above	54 (67.5)	26 (32.5)	80 (26.7)
Total	169 (56.3)	131 (43.7)	300 (100.0)

The levels of biochemical parameters (triglyceride, total cholesterol, VLDL-C, LDL-C, HDL-C and fasting blood sugar level) and the anthropometric parameters (waist circumference, BMI) did not differ significantly between the two sexes except for the waist-hip ratio. Also, systolic and diastolic blood pressure levels did not differ significantly (Table II).

**Table II:** Clinical biochemistry, blood pressure and anthropometric parameters by sex

Parameters	Male N=169	Female N=131	Total N=300
Triglycerides (mg/dl)	133.91 ± 6.31	125.17 ± 6.81	130.11 ± 4.66
Total cholesterol (mg/dl)	167.24 ± 3.14	169.71 ± 3.28	168.31 ± 2.27
VLDL-C (mg/dl)	26.79 ± 1.27	24.81 ± 1.36	25.93 ± 0.93
LDL-C (mg/dl)	99.58 ± 2.86	101.84 ± 3.06	100.56 ± 2.09
HDL-C (mg/dl)	40.86 ± 1.08	42.98 ± 1.06	41.78 ± 0.76
Fasting glucose (mg/dl)	89.89 ± 1.47	90.63 ± 2.29	90.21 ± 1.29
Waist circumference (cm)	76.82 ± 0.87	74.91 ± 1.04	75.99 ± 0.67
Systolic BP (mm/Hg)	130.04 ± 1.20	130.20 ± 1.49	130.11 ± 0.94
Diastolic BP (mm/Hg)	82.42 ± 0.83	83.21 ± 0.96	82.76 ± 0.63
Body mass index (kg/m^2^)	22.84 ± 0.27	22.66 ± 0.28	22.76 ± 0.19
Waist-hip ratio^*^	0.83 ± 0.01	0.80 ± 0.01	0.82 ± 0.01
Values are mean ± SE; ^*^P <0.05			

The overall presence of metabolic syndrome as per ATP-III criteria was 5.0 per cent. It was significantly higher among females (7.6%) as compared to males (2.9%). When ATP-III criteria was modified using waist circumference cut-offs recommended by Asia-Pacific guidelines, metabolic syndrome was observed in 9.3 per cent. It was 10.7 per cent among females and 8.2 per cent among males. The difference was not statistically significant (Table III). The Figure shows the prevalence of metabolic syndrome by sex and age group. In youngest age group (18-30 yr), none of the male subject had metabolic syndrome while 4 per cent females had it. In the age group of 31-50 yr, more females than males were affected (16.4% in females vs. 10.9% in males) while in older age group (> 50 yr), more males were affected than females (13.0% in males vs. 11.5% in females).

**Table III:** Proportion of adults with metabolic syndrome and individual parameters above threshold levels

Parameters(yr)	Sex		Total N = 300
	Male N = 169	Female N = 131	
Triglycerides (≥ 150 mg/dl)	57 (33.5)	34 (26.0)	91 (30.2)
HDL-C (mg/dl) (M ≥ 40; F≥50)^*^	85 (50)	92 (70.2)	177 (58.8)
Fasting glucose (≥ 110 mg/dl)	23 (13.5)	14 (10.7)	37 (12.3)
Blood pressure (SBP ≥ 130 and/or DBP ≥ 85 mm/Hg)	91 (53.5)	71 (54.2)	162 (53.8)
Waist circumference Int (M ≥ 102 cm; F ≥ 88 cm)^*^	6 (3.5)	23 (17.6)	29 (9.6)
Waist circumference Asia (M ≥ 90 cm; F ≥ 80 cm)^*^	28 (16.5)	37 (28.2)	65 (21.6)
Metabolic syndrome (ATP III criteria)	5 (2.9)	10 (7.6)	15 (5.0)
Metabolic syndrome Asia (ATP III criteria modified as per Asia-Pacific guidelines)	14 (8.2)	14 (10.7)	28 (9.3)
Figures in parentheses are percentages; ^*^P<0.05			

**Fig.**
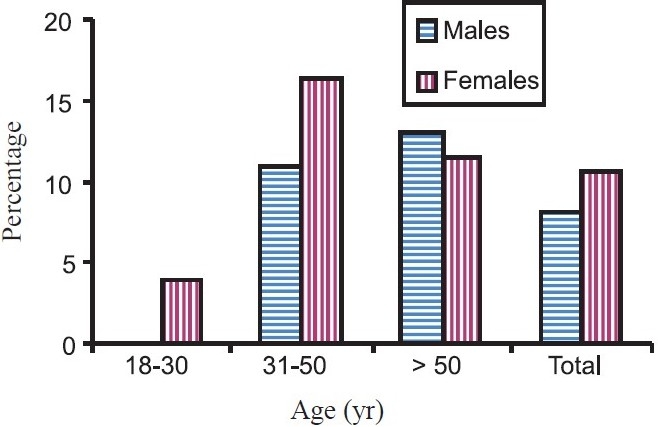
Prevalence of metabolic syndrome by age and sex.

ROC curve was plotted to find out the best cut-off of BMI to identify the individuals with metabolic syndrome. The best cut-off for BMI came out to be 23.32. Sensitivity of BMI at this cut-off was 0.75 (95% CI: 0.566-0.873), specificity was 0.685 (95% CI: 0.627-0.737). Likelihood ratio for BMI > 23.32 was 2.38 (95% CI: 1.8-3.1) and likelihood ratio for BMI ≤ 23.32 was 0.36 (95% CI: 0.19-0.69). The diagnostic odds ratio was 6.52 (95% CI: 2.67-15.9).

## Discussion

In the present study, metabolic syndrome was found in 9.3 per cent rural population by modified criteria as per Asia-Pacific guidelines. Traditional societies and population residing in and around rural areas are expected to have low prevalence as these are not exposed to modernization^8^. The ICMR task force collaborative study reported the prevalence of metabolic syndrome to be 30 per cent in urban areas of Delhi and 11 per cent in rural Haryana using ATP-3 criteria^20^. Mishra *et al*^9^reported 30 per cent prevalence among the urban slum population in Delhi. Ramchandran *et al*^21^, reported a prevalence of 41 per cent in urban area of Chennai using modified ATP-3 criteria among adults aged 20 to 75 yr^21^. They also reported that prevalence was higher in women than men (46.5 vs 36.4%) and in older people. Sarkar *et al*^8^reported 30-50 per cent prevalence in Bhutia tribe, with no rural-urban difference. Among the Toto tribe, the rural community prevalence was low 4-9 per cent^8^. The differences may be attributed to the difference in study areas, and the different definitions of metabolic syndrome used.

Higher abdominal fat (android obesity) is known to be a risk factor for hypertension, hypertriglyceridaemia, hyperinsulinaemia and diabetes^22^–^24^. The BMI though does not account the distribution of body fat, is recommended by the World Health Organization as the most useful epidemiological measure of obesity and is most commonly used to tract obesity and related diseases^25^,^26^. In Caucasian populations, the association between BMI and mortality is “J-shaped” and nadir of the curve between 18.5 - 25.0 kg/m^2^ is taken as healthy BMI^27^. Cut-off values of 25.0 and 30.0 kg/m^2^are taken for overweight and obesity respectively and also for identifying the risk of associated morbidities^28^. In an effort to map the epidemic of obesity and associated risk of co-morbidities, it has become common practice to use these cut-off values in different populations with the assumption that different ethnic groups have similar morbidity/mortality risk for the specific BMI level in absence of any such evidence^29^. Same is true for other anthropometric indicators. As evidence emerged from many studies about the higher susceptibility of Asians at lower BMI, international task force suggested lower cut-off values for them (23 and 25 kg/m^2^ for overweight and obesity respectively)^28^. Studies from urban India also suggested lower cut-off values of these anthropometric indicators^30^–^33^. Deshmukh *et al*^34^ also reported lower cut-offs of BMI (21.7 kg/m^2^for males and 21.2 kg/m^2^ for females) and waist circumference (72.5 cm for males and 65.5 cm for females) for detection of hypertension in rural Wardha. In the present study, BMI of 23.32 kg/m^2^ emerged out to be the best cut-off for prediction of risk of metabolic syndrome. Asia-Pacific guidelines also advocate the use of BMI > 23 kg/m^2^ as a definition of overweight in Asian populations.

To conclude, the presence of metabolic syndrome was seen in 5.0 per cent rural adults of Wardha by ATP-III criteria. It was 9.2 per cent when the cut-off for waist circumference was modified as per Asia-Pacific guidelines. BMI of 23.32 kg/m^2^ and higher predicts significant risk of metabolic syndrome in these study subjects. As the higher degree of error was considered for the calculation of sample size which has led to decrease in the power of the study, studies with larger sample size need to be conducted to validate the present findings.
